# Peptide translocation by the lysosomal ABC transporter TAPL is regulated by coupling efficiency and activation energy

**DOI:** 10.1038/s41598-019-48343-6

**Published:** 2019-08-15

**Authors:** Christoph Bock, Tina Zollmann, Katharina-Astrid Lindt, Robert Tampé, Rupert Abele

**Affiliations:** Institute of Biochemistry, Biocenter, Goethe University Frankfurt, Max-von-Laue Str. 9, 60438 Frankfurt am Main, Germany

**Keywords:** Enzyme mechanisms, Membrane proteins

## Abstract

The lysosomal polypeptide transporter TAPL belongs to the superfamily of ATP-binding cassette transporters. TAPL forms a homodimeric transport complex, which translocates oligo- and polypeptides into the lumen of lysosomes driven by ATP hydrolysis. Although the structure and the function of ABC transporters were intensively studied in the past, details about the single steps of the transport cycle are still elusive. Therefore, we analyzed the coupling of peptide binding, transport and ATP hydrolysis for different substrate sizes. Although longer and shorter peptides bind with the same affinity and are transported with identical K_m_ values, they differ significantly in their transport rates. This difference can be attributed to a higher activation energy for the longer peptide. TAPL shows a basal ATPase activity, which is inhibited in the presence of longer peptides. Uncoupling between ATP hydrolysis and peptide transport increases with peptide length. Remarkably, also the type of nucleotide determines the uncoupling. While GTP is hydrolyzed as good as ATP, peptide transport is significantly reduced. In conclusion, TAPL does not differentiate between transport substrates in the binding process but during the following steps in the transport cycle, whereas, on the other hand, not only the coupling efficiency but also the activation energy varies depending on the size of peptide substrate.

## Introduction

ATP binding cassette (ABC) transporters are part of the ABC protein superfamily and form the largest class of primary active transport proteins, which couple ATP hydrolysis with substrate transport. The substrate spectrum of ABC transporters ranges from small soluble molecules, hydrophobic drugs and lipids to large proteins^1^. ABC transporters include importers, exporters, channels, and regulators. Their basic architecture comprises two nucleotide-binding domains (NBDs) and two transmembrane domains (TMDs). In eukaryotes, the ABC transporters exist as full-transporters with all four domains combined in one polypeptide chain or as half-transporters composed of one TMD and one NBD. Consequently, half-transporters can either form homo- or heterodimers. Since many transporters are linked to human diseases, such as cystic fibrosis, Tangier disease, or Dubin-Johnson syndrome, elucidating the molecular mechanisms and functions of ABC transporters is of high interest^2,3^.

A representative ABC exporter is the half-transporter associated with antigen processing-like (ABCB9, TAPL), which translocates peptides into the lumen of lysosomes^4^. Besides its broad tissue distribution with highest expression in dendritic cells, macrophages, and Sertoli cells, TAPL orthologous are also found in invertebrates like *Caenorhabditis elegans* and even in plants^5–8^. The core complex of TAPL (coreTAPL), composed of 2 × 6 C-terminal transmembrane helices (TMHs) and two NBDs, forms homodimers and is fully active in ATP hydrolysis as well as in substrate translocation. However, coreTAPL is mistargeted to the plasma membrane since lysosomal targeting is encoded by the N-terminal, four TMHs comprising TMD0^9,10^. This domain is also important for the interaction with the lysosomal membrane proteins LAMP-1/2, increasing the half-life of the transporter^11^. TAPL displays a strictly unidirectional transport of cytosolic peptides ranging from 6- up to 59-mers into the lysosomal lumen with a substrate preference for peptides with positively charged or large hydrophobic N- and C-terminal residues^12–14^. TAPL can accumulate peptides more than 1000-fold against its gradient as demonstrated by dual color fluorescence burst analysis. However, peptide transport stops at a luminal concentration of 1 mM. In this trans-inhibition state, the luminal peptide potentially occupies the low affinity binding site and prevents the TMD to return back to the inward conformation^14^.

As postulated by Jardetzky^15^ and supported by several X-ray structures^16–18^ and cryogenic electron microscopy reconstructions^19,20^ as well as EPR studies^21,22^, ABC exporters alternate between an inward and outward open conformation. This movement is coordinated by ATP binding and hydrolysis in the NBDs. Two ATP molecules bind at the interface of the NBDs by contacting Walker A and B, as well as A-, Q-, and H-loop from one NBD and ABC signature (C-loop) from the opposite NBD. ATP-induced NBD dimerization is accompanied by a movement of the TMD from an inward- to an outward-facing conformation, mediated by complex interactions between coupling helices within the TMDs and a groove on the surface of the NBDs formed by the X- and Q-loop between the RecA-like and α-helical subdomains. Subsequently, substrate is released and ATP hydrolyzed, which eventually induces NBD dissociation and a conformational switch back into the inward-facing conformation^23^. This cycle is based on a coupling between solute transport and ATP hydrolysis. The transporter associated with antigen processing 1 and 2 (ABCB2/3, TAP1/2) shows such a strict coupling since ATP is only hydrolyzed when peptide is bound and transported^24^. However, other ABC exporters exhibit a less stringent coupling with high basal ATPase activity^25–29^. Thus, it seems that there is a gradual coupling between ATP hydrolysis and substrate transport. Numerous studies were performed with hydrophobic substrates for which quantitative transport assays are often not feasible. Moreover, for some of the studied ABC transporters, a solid quantitative and, thus, mechanistic analysis is even more delicate since detergents or lipids act as potential substrates. Therefore, TAPL is a perfect example for studying the transport cycle, as it exports easily trackable hydrophilic peptides of very different sizes.

In this study we determined thermodynamic and kinetic parameters of peptide binding and transport by TAPL and correlated them with ATP hydrolysis. We found that smaller and longer peptides bind with a similar affinity and are transported with identical K_m_ values. However, the transport rate is drastically reduced for longer peptides, which can be attributed to higher activation energy and differential coupling between ATP hydrolysis and substrate translocation. The high basal ATPase activity of TAPL is not stimulated by peptide transport, which is pointing to a strong uncoupling between ATP hydrolysis and peptide transport. Strikingly, uncoupling is not only dependent on the length of the transported peptide, but also on the type of nucleotide used for energizing the transport.

## Results

### Peptide binding

**Figure 1:**
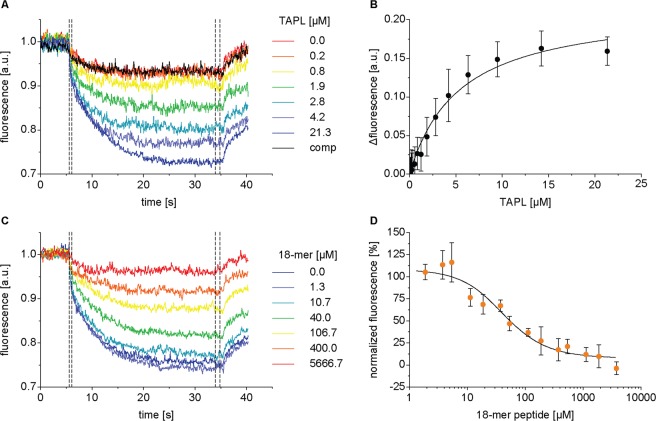
Peptide binding of TAPL by microscale thermophoresis. K_d_ of 9-mer (**A**,**B**) and K_i_ of 18-mer peptide (**C**,**D**) were determined by microscale thermophoresis at 22 °C. (**A**) Increasing concentrations of TAPL were titrated to fluorescently labeled 9-mer^Atto-655^ peptide (100 nM) resulting in thermophoresis based fluorescence changes exemplary shown by normalized microscale thermophoresis traces. Specific binding was demonstrated by competition with 4 mM unlabeled 9-mer peptide (comp) in the presence of 10 µM TAPL. (**B**) Mean fluorescence changes (equation 1) are plotted against the concentration of TAPL and fitted by a one-site binding model (equation 2) (K_d_ = 5.4 ± 0.8 µM; R^2^ = 0.98). (**C**) TAPL (10 µM) was incubated with 9-mer^Atto-655^ peptide (100 nM) and the interaction was inhibited with increasing concentrations of 18-mer peptide. (**D**) Normalized fluorescence changes were plotted against 18-mer peptide concentration, fitted by equation 3 resulting in a K_i_ value of 11.8 ± 3.6 µM (R^2^ = 0.95) considering competitor depletion. Dotted lines indicate areas used for determination of mean fluorescence values, error bars indicate SD of triplicate measurements.

To characterize the coupling between ATP hydrolysis and peptide translocation of TAPL, we step by step analyzed peptide binding and transport as well as ATP hydrolysis for a 9-mer peptide (RRYQKSTEL), a spatial challenging 18-mer peptide (RRYQKSTELRRYQKSTEL) and a branched 16-mer peptide (RYCK(K_8_)STEL) carrying a poly-lysine chain connected by isopeptide bonds. CoreTAPL, C-terminally fused to the fluorescent protein mVenus, was produced in *Pichia pastoris*, solubilized by dodecyl-β-D-maltoside, and purified via metal affinity chromatography. This coreTAPL construct features the same kinetic parameters as wt TAPL produced in Sf9 insect cells^12–14^. The equilibrium binding constant was determined by microscale thermophoresis where the thermophoretic behavior of Atto-655 labeled 9-mer (RRYC^Atto-655^KSTEL, 0.1 µM) with increasing concentrations of TAPL was detected by fluorescence changes. Applying a Langmuir 1:1 binding isotherm, a dissociation constant K_d_ of 5.4 ± 0.8 µM was revealed for the 9-mer peptide (Fig. 1A,B). The affinity for the 18-mer peptide was specified by competition experiments applying 0.1 µM RRYC^Atto-655^KSTEL and 10 µM TAPL. Taking ligand depletion into account, an inhibitory constant K_i_ of 11.8 ± 3.6 µM was obtained (Fig. 1C,D). In conclusion, the length of these two peptides does not influence peptide binding supporting the binding mode where only the N- and C-terminal residues are involved in recognition^12^.

### Peptide transport

**Figure 2:**
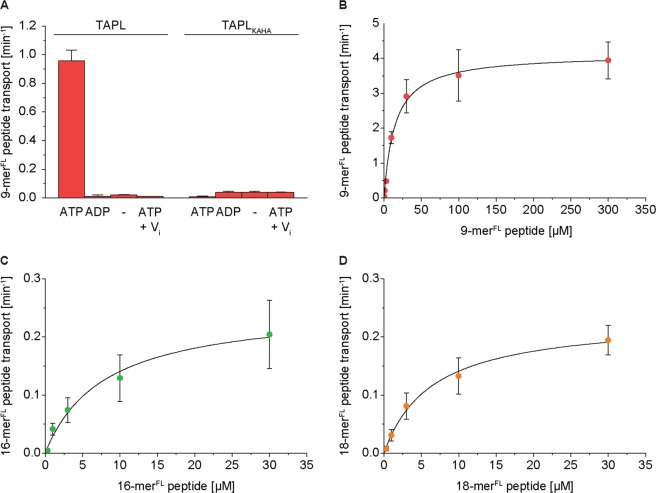
Peptide transport by TAPL. Peptide transport was performed with reconstituted TAPL or TAPL_KAHA_ (40:1 weight ratio of lipids to TAPL) for 15 min at 37 °C. (**A**) Proteoliposomes (0.5 mg/ml lipids) were incubated with 10 µM of 9-mer^FL^ peptide and 3 mM ATP, 3 mM ADP, without any nucleotide (−) or with 3 mM ATP in the presence of ortho-vanadate (V_i_, 500 µM). Peptide dependent Michaelis-Menten kinetics of 9-mer^FL^ (**B**), 16-mer^FL^ (**C**) and 18-mer^FL^ peptide (**D**) were performed in the presence of 3 mM ATP. Data were fitted by Michaelis-Menten equation (equation 4), K_m(Pep)_ and k_cat(Pep)_ values are depicted in Table 1. Steady-state conditions were demonstrated by time dependent peptide transport (Fig. S2). Transport was performed in triplicates with error bars indicating SD.

Next, we analyzed the transport kinetics of these peptides with TAPL reconstituted into proteoliposomes (Fig. S1). We applied steady-state conditions in which peptide transport increases linearly with time and is not inhibited by substrate depletion or by trans-inhibition (Fig. S2). As demonstrated for the fluorescein labeled 9-mer^FL^ peptide (RRYC^Fluorescein^KSTEL), peptide translocation is ATP dependent, specifically inhibited by ortho-vanadate and also lost upon mutation of the conserved lysine (K545) of the Walker A and histidine (H699) of the H-loop (TAPL_KAHA_) (Fig. 2). TAPL showed a Michaelis-Menten kinetic of peptide transport for 9-mer^FL^, 16-mer^FL^ and 18-mer^FL^ peptide with K_m(Pep)_ of 15 ± 2 µM, 8 ± 2 µM and 7 ± 1 µM, respectively. These K_m_ values agree well with the equilibrium binding constants (Table 1). Surprisingly, the turnover number k_cat(Pep)_ for the 9-mer^FL^ peptide (4.1 min^−1^) is 20-fold higher than for the 16-mer^FL^ and (0.25 min^−1^) and 18-mer^FL^ peptide (0.23 min^−1^).

**Table 1:** Kinetic parameters of peptide transport and ATP hydrolysis by TAPL.

Peptide^*^	ATP hydrolysis				Peptide transport		
	IC_50_ [µM]	Hill slope	k_cat(ATP)_ [min^−1^] ^#^	R^2^	K_m(Pep)_ [µM]	k_cat(Pep)_ [min^−1^]	R^2^
—	—	—	75 ± 4	—	—	—	—
9-mer	—	—	65 ± 7	—	15 ± 2	4.14 ± 0.11	0.99
16-mer	22 ± 1	−2.09 ± 0.73	22 ± 4	0.98	8 ± 2	0.25 ± 0.02	0.98
18-mer	15 ± 1	−0.86 ± 0.11	29 ± 1	0.97	7 ± 1	0.23 ± 0.01	0.99

*9-mer, 16-mer and 18-mer peptide were used to determine ATPase activity of TAPL; 9-mer^FL^, 16-mer^FL^ and 18-mer^FL^ peptide were used to determine peptide transport activity of TAPL.

^#^Rate constant of ATP hydrolysis under saturating peptide concentration taken from Fig. 4B.

**Figure 3:**
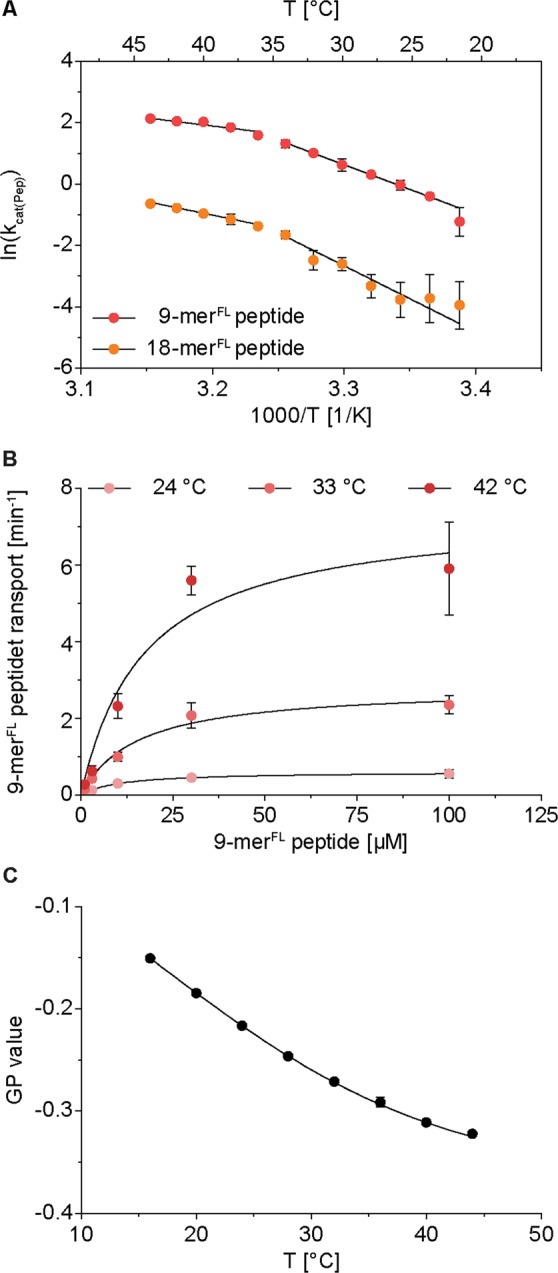
Arrhenius analysis of peptide transport. (**A**,**B**) To determine temperature dependent peptide transport, proteoliposomes (0.5 mg/ml lipids) containing TAPL (40:1 weight ratio of lipids to TAPL) were incubated with 3 mM ATP for 15 min between 22 and 44 °C. (**A**) Peptide transport was performed with 9-mer^FL^ peptide (100 µM) and 18-mer^FL^ peptide (25 µM). The natural logarithm of the rate constants was plotted against the reciprocal of the temperature and data were fitted with two linear regressions. The intersection of the two linear regressions for transport is at 36.3 °C. Activation energies were calculated by Arrhenius equation (equation 5) and are depicted in Table 2. (**B**) Michaelis-Menten kinetics of 9-mer^FL^ peptide (100 µM) transport at different temperatures were determined at steady-state conditions (Fig. S3) and fitted with equation 4. K_m(Pep)_ values of 10.8 ± 0.4 µM (R^2^ = 0.99), 15.3 ± 3.8 µM (R^2^ = 0.98) and 17.3 ± 7.8 µM (R^2^ = 0.95) were determined at 24 °C (light red), 33 °C (red) and 42 °C (dark red), respectively. (**C**) Membrane fluidity of proteoliposomes comprised of *E. coli* polar lipids and DOPC (7:3 (wt:wt)) was analyzed by generalized polarization (GP) of C-Laurdan at different temperatures (Fig. S4). Data were measured in triplicates and error bars indicate SD.

To find a reason for this discrepancy in transport rates, although all peptides are equally well recognized, we studied the activation energies of peptide transport. For this, we determined the temperature dependent transport rates under saturating peptide and ATP concentrations applying steady-state conditions (Fig. S3). We calculated the Arrhenius activation energy from the negative slope of the Arrhenius plots (Fig. 3A). For each temperature analyzed, the 9-mer^FL^ peptide showed a higher transport rate than the 18-mer^FL^ peptide. For both peptides, a biphasic temperature dependence was detected. Below 36 °C, activation energies of 134 kJ/mol and 178 kJ/mol were determined for the 9-mer and 18-mer peptide, respectively (Table 2). Above 36 °C, the activation energies decreased to 44 kJ/mol and 77 kJ/mol for the short and long peptide, respectively. Non-linear Arrhenius plots can indicate a temperature dependent change in the rate-limiting step of the transport cycle or can emerge from a temperature dependence of substrate affinity or from lipid phase transitions. Temperature dependent shifts in binding affinities were excluded since K_m_ values for peptide stayed constant for the complete temperature range (Fig. 3B). Therefore, the binding affinities seem to be unaffected in the temperature range analyzed. To study lipid phase transitions, we determined lipid organization in proteoliposomes by generalized polarization of C-Laurdan between 16 and 44 °C. However, the trend of the generalized polarization of C-Laurdan indicates that the lipid mixture of *Escherichia coli* polar lipids and 1,2-di(9-octadecenoyl)-*sn*-glycero-3-phosphocholine (DOPC) in a weight ratio of seven to three does not undergo a phase transition in the applied temperature regime (Figs 3C; S4). In summary, for both peptides the rate-limiting step in transport alters at 36 °C. The high activation energies at lower temperatures indicate large conformational changes involved in the rate-limiting step. At higher temperatures, the activation energies decrease drastically. Importantly, lower transport rates of the 18-mer peptide result from higher activation energies compared to the 9-mer peptide.

**Table 2:** Activation energies during transport by Arrhenius analysis.

Peptide	E_a_^*^ [kJ/mol]	R^2^	E_a_^#^ [kJ/mol]	R^2^
9-mer^FL^	44 ± 6	0.94	134 ± 15	0.99
18-mer^FL^	77 ± 5	0.98	178 ± 18	0.95

*Temperature between 36 and 44 °C.

^#^Temperature between 22 and 34 °C.

### ATP hydrolysis

**Figure 4:**
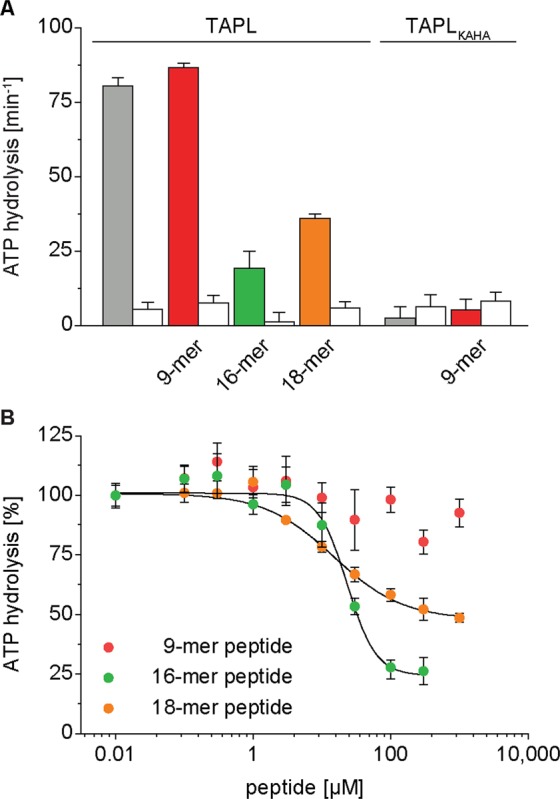
Peptide dependent ATPase activity. Proteoliposomes (0.5 mg/ml lipids) containing TAPL or TAPL_KAHA_ (40:1 weight ratio of lipids to TAPL) were incubated with 3 mM ATP for 15 min at 37 °C. ATP hydrolysis was determined by malachite green based assay in the absence or presence of 100 µM peptide (filled bar) or ortho-vanadate (500 µM, white bar) (**A**) or with increasing peptide concentrations (**B**). Hydrolysis rates of 16-mer and 18-mer peptide were fitted with equation 6. IC_50_ values, Hill coefficients and k_cat_ are depicted in Table 1. Steady-state conditions were demonstrated by time dependent ATP hydrolysis (Fig. S5). All experiments were performed in triplicates, error bars indicate SD.

To correlate peptide transport with ATP hydrolysis, we determined ATP hydrolysis rates of TAPL reconstituted in liposomes in the presence and absence of short and long peptides. In the absence of peptides, TAPL revealed a basal ATPase activity of approximately 70 min^−1^, which was blocked by the ABC transporter specific ATPase inhibitor ortho-vanadate (Fig. 4A). In the presence of the transport inactive mutant TAPL_KAHA_, only background ATP hydrolysis was observed, confirming that TAPL specific ATP hydrolysis was detected. The basal ATP hydrolysis rate was not altered by addition of the 9-mer peptide RRYQKSTEL, even at high concentrations of 100 µM, implying the absence of a communication between the nucleotide- and peptide-binding sites. Interestingly, ATP hydrolysis was reduced in the presence of the spatial challenging 18-mer peptide by 50% and the branched 16-mer peptide by 75%. With increasing 9-mer peptide concentrations, ATP hydrolysis of TAPL was not significantly influenced. In contrast, ATP hydrolysis decreased sigmoidal for the longer peptides with IC_50_ value of 22 µM for the 16-mer peptide and 15 µM for the 18-mer peptide, which fits to the peptide affinity (Fig. 4B). Kinetics were recorded under steady-state conditions in which peptide accumulation in proteoliposomes is far below saturation (Figs S2,S5). Therefore, inhibition of ATP hydrolysis does not result from trans-inhibition, which is reached at 1 mM of the 9-mer peptide^14^. Combining the rate of ATP hydrolysis under saturating peptide concentrations with the turnover rate of peptide transport, a ratio of 20 and 100 ATP per translocated 9-mer and 16-mer or 18-mer peptide, respectively, is calculated. In conclusion, ATP hydrolysis is uncoupled from peptide transport.

### Coupling ATP hydrolysis and peptide transport

To decipher the coupling between nucleotide hydrolysis and peptide transport, we intended to reduce futile hydrolysis cycles without translocating peptides. Assuming a kinetic effect where ATP binding and subsequent ATP hydrolysis is much faster than peptide binding, a more stoichiometric coupling should be obtained with decreasing ATP concentrations. Therefore, we determined Michaelis-Menten kinetics of nucleotide hydrolysis and peptide transport with decreasing nucleotide concentrations. For sensitivity reasons, we switched from the colorimetric malachite green based assay to a radioactive based assay. In this assay, the hydrolysis rates are about twice that of the former one. Besides ATP, we used also GTP to analyze coupling between hydrolysis and peptide transport since it was shown before that GTP fuels peptide transport of TAPL to a lower extend than ATP^12^. The mechanism of energizing peptide transport by GTP seems to be similar to ATP since GTPase activity of TAPL was not stimulated over basal activity in the presence of 9-mer peptide and transport as well as GTP hydrolysis were inhibited by ortho-vanadate (Fig. S6). Moreover, background GTPases did not interfere with the analysis as demonstrated by the ATPase inactive mutant TAPL_KAHA_. In the presence of 3 mM GTP, the K_m(pep)_ value for the 9-mer transport (4.4 µM, Fig. S7) is similar to that in the presence of ATP, and thus the type of purine base does not affect peptide recognition.

**Figure 5:**
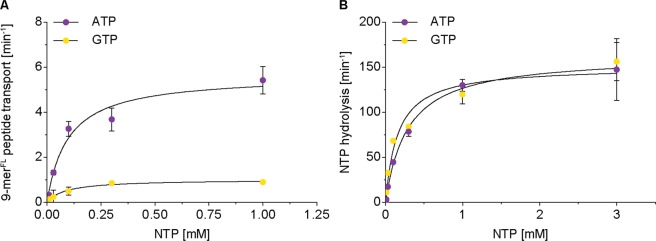
NTP dependent peptide transport and hydrolysis of TAPL. Peptide transport was performed with proteoliposomes (0.5 mg/ml) containing TAPL (40:1 ratio of lipids to TAPL) for 15 min at 37 °C in dependency of ATP and GTP under steady-stated conditions. (**A**) NTP dependent peptide transport was performed with 9-mer^FL^ peptide (100 µM). (**B**) NTP hydrolysis was analyzed in the presence of 9-mer peptide (100 µM) by radioactive based assay. Data were fitted by Michaelis-Menten equation (equation 4), K_m(NTP)_ and k_cat(NTP)_ values are listed in Table 3. Experiments were performed in triplicates, error bars indicate SD.

Subsequently, we performed Michaelis-Menten kinetics of 9-mer peptide (100 µM) transport using increasing ATP and GTP concentrations. The Michaelis-Menten constant for peptide transport was the same for ATP (K_m(ATP)_ = 97 µM) and GTP (K_m(GTP)_ = 87 µM), implicating similar binding affinities for both nucleotides (Fig. 5A, Table 3). However, the turnover number of peptide transport with ATP (k_cat(Pep)_ = 5.7 s^−1^) was 6-fold higher than with GTP (k_cat(Pep)_ = 1.0 s^−1^). Additionally, we quantified the nucleotide hydrolysis kinetics of ATP and GTP in the presence and absence of the 9-mer peptide. Again, the K_m(NTP)_ values for ATP and GTP hydrolysis are comparable and, unexpectedly, the turnover numbers for ATP and GTP hydrolysis are similar, regardless of peptide availability (Figs 5B, S8 Table 3). Correlating the rates of nucleotide hydrolysis and peptide transport, a constant ratio of 20:1 for ATP and 100:1 for GTP was found, regardless of the nucleotide concentration applied. Therefore, TAPL seems to have several cycles of futile nucleotide hydrolysis before a single peptide is translocated. Moreover, we assume a subtle difference in the cross-talk between the nucleotide binding domain and the transmembrane domain, which is caused by the type of purine base.

**Table 3:** NTP sensitivity of TAPL

	NTP hydrolysis*			Peptide transport^#^		
	k_cat(NTP)_ [min^−1^]	K_m(NTP)_ [µM]	R^2^	k_cat(Pep)_ [min^−1^]	K_m(NTP)_ [µM]	R^2^
ATP^(+)^	151 ± 10	160 ± 43	0.97	5.7 ± 0.5	97 ± 29	0.97
GTP^(+)^	163 ± 4	289 ± 22	0.99	1.0 ± 0.1	87 ± 19	0.98
ATP^(−)^	227 ± 12	153 ± 31	0.98	—	—	—
GTP^(−)^	175 ± 5	227 ± 24	0.99	—	—	—

*NTP hydrolysis in the presence^(+)^ or absence^(−)^ of 9-mer peptide.

^#^Peptide transport in the presence of 9-mer^FL^ peptide.

**Figure 6:**
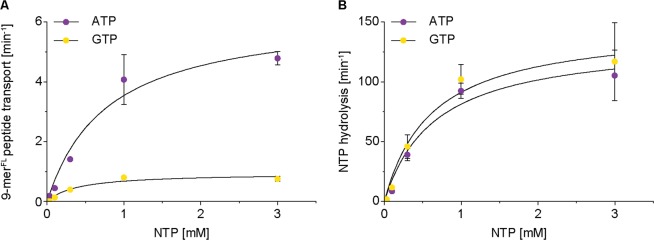
NTP dependent peptide transport and hydrolysis of TmrAB. Proteoliposomes (0.5 mg/ml lipids) containing TmrAB (40:1 weight ratio of lipids to TmrAB) were incubated under steady-state conditions (Fig. S11) for 10 min at 68 °C in dependency of ATP and GTP in the presence of 9-mer^FL^ peptide (30 µM). NTP dependent peptide transport (**A**) and radioactive NTPase assays (**B**) were performed under steady-stated conditions. Data were fitted by Michaelis-Menten equation (equation 4), K_m(NTP)_ and k_cat(NTP_) values are shown in Table 4. Experiments were performed in triplicates and error bars indicate SD.

To substantiate these observations, we analyzed nucleotide dependent peptide transport of the heterodimeric ABC transporter TmrAB from the thermophilic bacterium *Thermus thermophilus*^30^. TmrAB was reconstituted under identical conditions (Fig. S9), but nucleotide hydrolysis and peptide transport were measured at 68 °C (Fig. S10). Interestingly, in terms of hydrolysis, TmrAB does not differentiate between ATP and GTP but, as TAPL, shows a 6-fold decreased transport rate for GTP compared to ATP (Fig. 6, Table 4). Again, there is no stoichiometric coupling between nucleotide hydrolysis and peptide transport. In summary, the eukaryotic homodimeric TAPL complex as well as the prokaryotic heterodimeric transport complex TmrAB do not differentiate between ATP and GTP in respect of hydrolysis. Both transport complexes show a high degree of futile hydrolysis cycles, whereas GTP is even less efficient for transport than ATP.

**Table 4:** NTP sensitivity of TmrAB.

	NTP hydrolysis			Peptide transport		
	k_cat(NTP)_ [min^−1^]	K_m(NTP)_ [µM]	R^2^	k_cat(Pep)_ [min^−1^]	K_m(NTP)_ [µM]	R^2^
ATP	135 ± 13	661 ± 176	0.98	6.3 ± 0.6	782 ± 213	0.98
GTP	148 ± 12	617 ± 144	0.98	1.0 ± 0.1	391 ± 136	0.96

## Discussion

The thermodynamic and kinetic studies of TAPL presented herein show that TAPL binds and transports smaller and larger peptides with the same affinity and Michaelis-Menten constant. However, transport rate of the longer peptides is strongly reduced, which is accompanied by higher activation energy. Strikingly, the basal ATPase activity is not stimulated by any type of peptide, but is inhibited by longer ones. The stoichiometry between ATP hydrolysis and peptide transport increases with peptide length, but stays constant over the tested ATP concentration range. Remarkably, GTP displays the same kinetic parameters as ATP concerning hydrolysis, but differs significantly in respect of peptide transport.

To determine peptide binding affinity of TAPL, we applied several assays. However, all heterogeneous binding assays were unsuccessful since the dissociation rate constant is too fast. Microscale thermophoresis was the preferential method because the reaction volume was minimal compared to fluorescence anisotropy and gave identical results. Binding affinities of the 9-mer and 18-mer peptide are the same and fit well with the binding motif confined to the first N-terminal and last C-terminal residues^12,13^. In comparison, the closely related heterodimeric ER-resident antigen transport complex TAP recognizes the first three N-terminal and the last C-terminal residues of the peptides and binds therefore the 9-mer peptide with a 100-fold higher affinity^31,32^. Unfortunately, the stoichiometry of peptide binding to the TAPL complex cannot be addressed by microscale thermoporesis. It is conceivable that homodimeric TAPL forms two identical peptide binding sites, in contrast to heterodimeric TAP, which binds only one peptide^24^.

Despite the same binding affinity and Michaelis-Menten constant of the 9-mer and 18-mer peptide, they differ significantly in their turnover numbers. Several steps in the transport cycle feature different kinetics for both peptides since the activation energy of the rate-limiting step varies below and also above 36 °C between the 9-mer and 18-mer peptide. The large activation energy for both peptides below 36 °C indicates extensive structural rearrangements of TAPL. The rate-limiting step at higher temperature is accompanied by a lower activation energy reflecting a further step in the transport cycle. Such a low activation energy for substrate translocation was also observed for the secondary active glycerol-3-phosphate transporter (GlpT) although large conformational transitions occur here^33^. This discrepancy can be rationalized if the substrate-bound transporter adopts a conformation in a rapid process that allows peptide release with minor structural rearrangements. We speculate that the structural rearrangement, which is rate-limiting below 36 °C, is the step from the inward-facing to the occluded conformation of TAPL, whereas the rate-limiting step at higher temperature is the TMD opening to the lumenal site. The activation energy of both steps in the transport cycle is 30–40 kJ/mol higher for the 18-mer peptide compared to the 9-mer peptide, which could result from additional structural rearrangements of the peptide or within TAPL to form the occluded binding pocket.

In contrast to the heterodimeric TAP complex, which shows a strict coupling between peptide transport and ATP hydrolysis^34^, homodimeric TAPL depicts a high basal ATPase activity without any stimulation by substrate. Other ABC transporters like P-glycoprotein have a biphasic substrate dependent ATPase activity^28^. P-gp possesses a basal ATPase activity, which is stimulated at low substrate concentrations, and decreased at higher substrate concentration by trans-inhibition. Basal ATPase activity of MRP1 is 3-fold stimulated by its substrate leucotriene C_4_, which forms contacts to both halfs of the TMD and therefore brings the NBDs closer together^19^. Interestingly, Pdr5 also has high basal ATPase activity without any substrate stimulation, but with a substrate concentration dependent inhibition, most likely also induced by trans-inhibition^29^. At first glance, TAPL behaves like Pdr5 since ATPase activity of TAPL decreases with increasing concentrations of the 18-mer peptide. However, on closer examination, TAPL is clearly different since the peptide accumulation in proteoliposomes was in the linear range under the applied conditions, and a trans-inhibition of TAPL can thus be excluded. In addition, the 9-mer peptide did not inhibit basal ATP hydrolysis of TAPL and showed a 20-fold faster and a 5-fold more efficient transport than the 18-mer peptide, as reflected in the stoichiometry between ATP hydrolysis and peptide transport. The ATP-to-peptide stoichiometry was not dependent on the ATP concentration as reported for the maltose permease. In MalFGK_2_, the ratio shifts from 1.4 to 17 ATP per translocated maltose with increasing ATP concentration due to kinetics of ATP binding^35^. Therefore, we postulate that TAPL is a highly uncoupled ABC transporter in which ATP binding and hydrolysis are rarely associated with the conformational transition of the TMD from the inward to the outward open conformation, even if the peptide is bound. Structurally, such futile ATP hydrolysis can be rationalized by two scenarios: 1) Only the NBDs dimerize and the TMDs stay in their inward open conformation or 2) the TMDs adopts the occluded state. We prefer the scenario in which NBDs’ dimerization is uncoupled from TMDs’ movements, as observed for the macrolide transporter McjD by single-molecule Förster resonance energy transfer studies^36^. Data from pulsed EPR studies on P-gp suggest that in the presence of Zosuquidar, which inhibits transport but stimulates ATP hydrolysis, NBDs can dimerize although the TMDs are still in the inward facing conformation^37^.

Assuming that two ATP molecules are hydrolyzed per transport cycle regardless of the length of the peptide, the coupling between ATP hydrolysis and peptide transport is diminished. Likewise, the kinetics of a single translocation step are decreased for the 18-mer peptide in comparison to the 9-mer peptide. Since the activation energies of both identified steps are higher in the 18-mer peptide transport cycle than for the 9-mer peptide, the probability of futile ATP hydrolysis is increased for the 18-mer peptide, resulting in a higher ATP-peptide stoichiometry. Additionally, the translocation of a single 18-mer is decelerated again, which is reflected in the higher activation energy. In conclusion, ATP hydrolysis is uncoupled from peptide transport, while the degree of uncoupling depends on the length of the peptide. How the size of the peptides influences rate of ATP hydrolysis and peptide transport is not solved. However, it can be speculated that the additional space filled by the longer peptides influences the communication between the TMD and the NBDs. Also the overall structures of P-gp bound to Taxol, which is a transport substrate, and to the inhibitor Zosuquidar are very similar, there are subtle changes originating from the substrate binding site and are transmitted and amplified via TMHs to the NBDs^38^. In ABCG2, the introduction of a more bulky residue in the substrate binding site strongly increased ATPase activity whereas it abrogated transport of estrone-3-sulfate^39^.

Homodimeric TAPL with two consensus ATP-binding sites as well as heterodimeric TmrAB with one degenerate and one consensus ATP-binding site do not differentiate between ATP and GTP. Both nucleotides show the same K_m_ and k_cat_ values in respect of NTP hydrolysis. However, peptide transport is significantly reduced for GTP compared to ATP. NTP dependent transport was found also for other ABC transporters. PatA/PatB from *Streptococcus pneumonia* strongly prefers GTP over ATP for transport and NTP hydrolysis at 37 °C^40^. The full-length ABC transport Pdr5 from yeast hydrolyzes GTP and even UTP, but with highest efficiency ATP^41^. Interestingly, ATPase activity of Pdr5 is inhibited by clotrimazole, whereas GTP and UTP hydrolysis is not affected. Moreover, GTP energizes clotrimazole transport. It was proposed that the different NTP binding and hydrolysis kinetics influence the equilibration time of the transporter with its transport substrate and therefore enlarge selectivity. For TAPL and TmrAB it looks different since GTP and ATP hydrolysis rates are identical. We therefore hypothesize that besides the transport substrate also the nucleotide is essential for the coupling between NTP hydrolysis and conformational changes of the TMD. In conclusion, TAPL does not regulate the transport efficiency by peptide binding, but by subsequent steps in the transport cycle, which influence the coupling efficiency and the translocation rate.

In conclusion, the length of the peptide and the type of nucleotide are sensed in several steps of the transport cycle. It will be a challenge to identify the sensors engaged in the differential coupling of ATP hydrolysis and peptide transport at a molecular level.

## Materials and Methods

### Antibodies

For detection by immunoblotting the following antibodies were used: rabbit anti-TAPL^12^ and mouse anti-His (monoclonal, HIS-1; Sigma-Aldrich now Merck); horseradish peroxidase-conjugated goat anti-rabbit (polyclonal; BD Pharmingen) and goat anti-mouse (polyclonal A2554; Sigma-Aldrich now Merck).

### Constructs and cloning

Human coreTAPL (Q9NP78 UniProt, amino acid 143–766) fused C-terminally to mVenus and TmrAB (Q72J05, Q72J04 UniProt) from *Thermus thermophilus* were used as described^14,30^. CoreTAPL_HAKA_ was generated from human coreTAPL fused to mVenus in pPICZ-C^14^ by Ligase Chain Reaction using the primer pair K545A 5′-GGATGTTGACACAGGAGCTAGCC CCACTGC-3′, 5′-CCTCGGGCAGTGGGGCTAGCTCCTGTG-3′ and the primer pair H699A 5′-GTGCTCCACGGTGCTCAGCCGAGCCGCGATGATG-3′, 5′-CACGGTACTCATCATCGC GGCTCGGCTGAGCAC-3′. Transformation of the protease-deficient *Pichia pastoris* strain SMD1168-His^+^ was performed by electroporation as described^42,43^ (1.5 kV, 25 µF, 200 Ω, Gene Pulser X Cell, BioRad), using 30 µg linearized DNA. Transformant with highest expression was selected by YFP fluorescence^42,44,45^.

### Expression, purification and reconstitution

CoreTAPL fused C-terminally to mVenus was produced in *P. pastoris* by large scale expression in a 7.5 liter Labfors4 reactor (Infors HT, Einsbach, Germany) by fed batch method and cell pellets were stored at −80 °C^14,44^. CoreTAPL_HAKA_ fused C-terminally to mVenus was produced in *P. pastoris* in Erlenmeyer flasks^42^. TmrAB from *T. thermophilus* was expressed in *Escherichia coli* and cell pellets were stored at −80 °C^30^. CoreTAPL and TmrAB were purified and reconstituted (40:1 (wt/wt) lipid to protein) as described into unilamellar liposomes composed of *E. coli* polar lipids and DOPC (7:3 (wt/wt); Avanti Polar Lipids), which were ultrasonicated, five times frozen and thawed and extruded through a 400 nm polycarbonate filter (Avestin, Mannheim, Germany)^13,14,30^. Proteoliposomes were snap frozen in liquid nitrogen and stored at −80 °C. For TmrAB purification, the following alterations were made: cells were sonicated instead of using French press, and neither tobacco etch virus protease treatment to remove the His_10_-tag on TmrA, nor additional size exclusion chromatography have been performed. Concentration of coreTAPL and coreTAPL_KAHA_ was determined by specific absorption of mVenus (ε_515_ = 45.950 M^−1^cm^−1^). For calculation of TmrAB concentration ε_280_ = 159.630 M^−1^cm^−1^ was applied. Purity of TAPL, TAPL_KAHA_ and TmrAB was analyzed by SDS-PAGE (10%) followed by Coomassie staining (InstantBlue^TM^; Expedeon) or immunoblotting with TAPL- or His-tag specific antibody, respectively.

### Peptide labeling and purification

9-mer peptides (RRYQKSTEL, RRYCKSTEL), 18-mer peptides (RRYQKSTELRRYQKSTEL, RRYQKSTELRRYCKSTEL) (Charité, Medical University, Berlin) and branched 16-mer peptide (RYCK(K)_8_STEL, octa-lysine peptide coupled by isopeptide bonds) (EMC microcollections, Tübingen) were synthesized by solid phase synthesis. For labeling, single cysteine containing peptides were dissolved in PBS (10 mM Na_2_HPO_4_, 2 mM KH_2_PO_4_, 137 mM NaCl, 2.7 mM KCl, pH 6.5) supplemented with 20% (v/v) dimethylformamide (DMF) and incubated with a 1.2-fold molar excess of 5-iodoacetamidofluorescein (Sigma-Aldrich now Merck) or Atto-655-maleimide (ATTO-TEC) dissolved in DMF for at least 2 h at room temperature in an overhead rotator. Labeled peptides were purified by reversed-phase C_18_-HPLC (PerfectSil 300 ODS C18, 5 µm, 250 × 10 mm; MZ Analysentechnik) using a linear gradient from 5 to 100% (v/v) acetonitrile supplemented with 0.1% trifluoroacetic acid. Isolated fluorescent peptides were snap-frozen in liquid nitrogen and freeze-dried. Peptide identity and purity were verified by mass spectrometry and reversed-phase C_18_-HPLC (JASCO Germany). Peptide concentrations were determined by specific absorption (Fluorescein: ε_492_ = 75,000 M^−1^ cm^−1^ at pH 9.0 and Atto-655: ε_663_ = 125,000 M^−1^ cm^−1^ at pH 7.4).

### Peptide binding by microscale thermophoresis

To determine peptide affinity, 100 nM of the 9-mer^Atto-655^ peptide were incubated with increasing TAPL concentrations in purification buffer (20 mM HEPES, 140 mM NaCl, 0.05% (w/v) DDM, 15% (v/v) glycerol, pH 7.5). Microscale thermophoresis measurements were performed in hydrophilic capillaries (6 µl) at 22 °C on a Monolith NT.155Pico (NanoTemperTechnologies). Heating was induced for 30 s by an infrared laser and TAPL dependent changes in fluorescence of the 9-mer^Atto-655^ peptide were detected using 1% red LED power for excitation (λ_ex/em_: 598-642 nm/657–720 nm). Fluorescence change ΔF was calculated from ratio of the mean intensities at the beginning (5.6–6.1 s) and at the end of heating (34–34.9 s):

1 $${\rm{\Delta }}F=\frac{{F}_{hot}}{{F}_{cold}}$$

ΔF in the absence of TAPL was subtracted from the ΔF in the presence of TAPL and the dissociation constant K_d_ was determined by fitting data with a one-site binding model (equation 2).

2 $${\rm{\Delta }}{F}_{cor}={\rm{\Delta }}{F}_{max}\times \frac{[TAPL]}{{K}_{d}+[TAPL]}$$

ΔF_cor_ represents the background corrected mean intensity fluorescence ratio, ΔF_max_ the maximum mean intensity fluorescence ratio and K_d_ the equilibrium dissociation constant.

To determine the affinity of the unlabeled 18-mer peptide by competition, 100 nM of 9-mer^Atto-655^ peptide were incubated with 10 µM TAPL and titrated with increasing concentrations of unlabeled 18-mer peptide. For determination of the dissociation constants K_i_ of the unlabeled peptide, competitor depletion was considered^46^.

3 $$Y={F}_{min}+({F}_{max}-{F}_{min})\times (\frac{2\times \sqrt{({a}^{2}-3b)}\times \,\cos (\frac{d}{3})-a}{3\times {K}_{d}+(2\times \sqrt{({a}^{2}-3b)}\times \,\cos (\frac{d}{3})-a})$$

With

$$a={K}_{d}+{K}_{i}+{L}_{I}+{L}_{R}-R$$

$$b={K}_{i}\times ({L}_{R}-R)+{K}_{d}\times ({L}_{I}-R)+{K}_{d}\times {K}_{i}$$

$$c={K}_{d}\times {K}_{i}\times R$$

$$d={\cos }^{-1}(\frac{-2\times {a}^{3}+9ab-27c}{2\times \sqrt{{({a}^{3}-3b)}^{3}}})$$

Y is the fluorescence signal in dependence of increasing concentration of unlabeled peptides. F_max_ is the signal in the absence of unlabeled peptides and F_min_ when all labeled peptides are competed. K_d_ is the dissociation constant of the labeled reporter peptide, L_R_ the concentration of the labeled reporter peptide, R the concentration of TAPL, L_I_ the concentration of the unlabeled peptide and K_i_ the affinity constant of the unlabeled competitor peptide.

### Peptide transport

Peptide transport was performed with 50 µl of proteoliposomes (0.5 mg/ml lipids) containing TAPL or TmrAB (40:1 (wt/wt) lipid to protein) in transport buffer (20 mM HEPES, 107 mM NaCl, 3 mM MgCl_2_, 5% (v/v) glycerol, pH 7.5) in the presence of fluorescein labeled 9-mer^FL^, 16-mer^FL^ and 18-mer^FL^ peptide. For concentrations higher than 30 µM, 9-mer^FL^ peptide was supplemented with unlabeled 9-mer peptide. Transport was started by addition of NTP or H_2_O for background determination and stopped by adding 200 µl of ice-cold stop buffer (PBS, 10 mM EDTA, pH 7.5). Transport of TAPL was monitored for 15 min at 37 °C unless stated different, whereas transport of TmrAB was always investigated for 10 min at 68 °C. Proteoliposomes were collected on micro-filter plates (MultiScreen plates, Durapore membrane, 0.65 µm pore size; Millipore now Merck) preincubated with 0.3% (w/v) polyethylenimine. Filters were washed three times with 250 µl of ice-cold stop buffer and incubated for 10 min in elution buffer (PBS, 1% SDS, pH 7.5). Amount of transported peptide was quantified using a microplate reader (CLARIOstar, BMG LABTECH) at λ_ex/em_ = 483/530 nm. Samples containing TAPL were heated for 10 min at 95 °C prior to quantification to exclude interference of mVenus fluorescence. Peptide dependent Michaelis-Menten transport kinetics were analyzed by fitting background corrected data with

4 $$v={v}_{max}\times \frac{[S]}{{K}_{m(Pep)}+[S]}$$

where v represents the concentration dependent transport rate, v_max_ the maximal transport rate, [S] the respective substrate concentration and K_m(Pep)_ the Michaelis-Menten constant. Arrhenius analysis of peptide transport was performed between 22 and 44 °C at saturating peptide concentrations. The logarithmic value of the rate constant of peptide transport was plotted against the reciprocal value of the temperature and fitted with

5 $$ln({k}_{cat(Pep)})=ln(A)\times \frac{-{E}_{a}}{R}\times \frac{1}{T}$$

where A represents the pre-exponential factor, R the gas constant (8.314 J*mol^−1−1^*K^−1^), T the temperature and E_a_ the activation energy.

### NTP hydrolysis

ATP and GTP hydrolysis of reconstituted TAPL and TmrAB were analyzed by malachite green based colorimetric assay^47^ or by a radioactive based assay. For TAPL, nucleotide hydrolysis was analyzed for 15 min at 37 °C unless stated different, whereas hydrolysis activity of TmrAB was investigated for 10 min at 68 °C. The malachite green based assay was performed in 25 µl of NTPase buffer (20 mM HEPES, 107 mM NaCl, 5 mM NaN_3_, 1 mM Ouabain, 50 µM EGTA, 3 mM MgCl_2_, 5% (v/v) glycerol, pH 7.5) containing proteoliposomes (0.5 mg/ml). The reaction was started by the addition of NTP. Background and autohydrolysis were determined with 500 µM ortho-vanadate or in the absence of protein, respectively. Hydrolysis was stopped by the addition of 20 mM H_2_SO_4_, supplemented with malachite green solution (3 mM malachite green, 0.2% (v/v) Tween 20, 1.5% (w/v) ammoniummolybdate) and absorption was detected after 10 min incubation at room temperature at 620 nm using a microplate reader (CLARIOstar, BMG LABTECH). IC_50_ values for peptides were determined by

6 $$v={v}_{min}+\frac{{v}_{max}-{v}_{min}}{1+{10}^{(LOGI{C}_{50}-[I])\cdot n}}$$

where v represents the peptide inhibitor [I] dependent hydrolysis rate, v_max_ the maximal hydrolysis rate in the absence of peptide, v_min_ the minimal hydrolysis rate at saturating peptide concentrations and n the Hill slope. IC_50_ represents the half maximal inhibitory peptide concentration.

The radioactive based assay was performed in 10 µl of NTPase buffer containing proteoliposomes (0.5 mg/ml) and NTP supplemented with tracer amounts of [γ-^32^P]-NTP (PerkinElmer) in the presence or absence of 100 µM 9-mer peptide for TAPL and 30 µM 9-mer^FL^ peptide for TmrAB. Autohydrolysis was determined in the absence of proteoliposomes. 1 µl of reaction mixture was spotted onto PEI cellulose plates (TLC PEI Cellulose F, Millipore now Merck). Inorganic phosphate and NTP were separated by thin layer chromatography using an equimolar mixture (0.8 M) of LiCl and acetic acid. ^32^P_i_ and [γ-^32^P]-NTP were quantified by autoradiography (PMI-Personal Moleculare Imager, Bio-Rad) and the amount of inorganic phosphate was calculated from the ratio of both signals. NTP-dependent Michaelis-Menten kinetics were determined by equation 4, where v represents the concentration dependent NTP hydrolysis rate, v_max_ the maximal NTP hydrolysis rate, [S] the substrate concentration and K_m(NTP)_ the Michaelis-Menten constant.

### Membrane fluidity

Membrane fluidity was analyzed by fluorescence spectroscopy with the membrane probe C-Laurdan (2d probes)^48,49^. C-Laurdan was added to proteoliposomes (33 µg/ml lipids) in a 1:500 molar ratio (C-Laurdan to phospholipids) and emission spectra were recorded between 400 and 600 nm after excitation at 375 nm for temperatures between 16 and 44 °C (Fluoromax-4 Spectrofluorometer, Jobin Yvon, with TC425Temperature Control system and Ext-440 Liquid Cooling System, Technology, Quantum Northwest and Koolance). Spectra were corrected for background from scattered light in the absence of C-Laurdan. The generalized polarization (GP) value as a relative measure for membrane order is calculated from the contribution of its blue (I_Ch1_:400–460 nm) and red (I_Ch2_: 470–530 nm) emission bands:

7 $$GP=\frac{{I}_{Ch1}-{I}_{Ch2}}{{I}_{Ch1}+{I}_{Ch2}}$$

## Supplementary information

Supplementary information
