# Supplementary material for: Peptide translocation by the lysosomal ABC transporter TAPL is regulated by coupling efficiency and activation energy
Source: Sci Rep. 2019 Aug 15;9:11884. doi: 10.1038/s41598-019-48343-6 (PMC6695453; doi:10.1038/s41598-019-48343-6)
Supplement: Supplementary file 1 — Supplementary information [file 41598_2019_48343_MOESM1_ESM.pdf]

Supplementary information

Peptide translocation by the lysosomal ABC transporter TAPL is regulated by coupling efficiency and activation energy

Christoph Bock[1], Tina Zollmann[1], Katharina-Astrid Lindt[1], Robert Tampé[1], Rupert Abele[1]*

[1] Institute of Biochemistry, Biocenter, Goethe University Frankfurt, Max-von-Laue Str. 9, 60438 Frankfurt, Germany

* Correspondence should be addressed to R.A.: abele@em.uni-frankfurt.de

Keywords: ABCB9, Arrhenius activation energy, GTP hydrolysis, peptide binding, transport kinetics

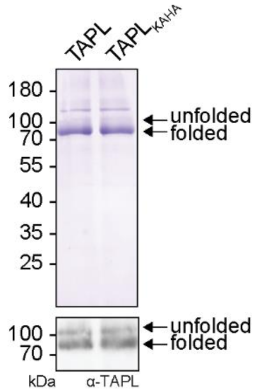

Figure S1: Purity of TAPL

TAPL and TAPL$_{KAHA}$ reconstituted in proteoliposomes were analyzed by SDS-PAGE (10%) followed by Coomassie staining (top, 2 µg protein per lane) or immunoblotting with TAPL specific antibody (bottom, 0.2 µg protein per lane). TAPL is split in two bands depicting unfolded and folded mVenus. Original, uncropped Coomassie stained SDS-PAGE and immunoblots are shown in supplementary information page 13.

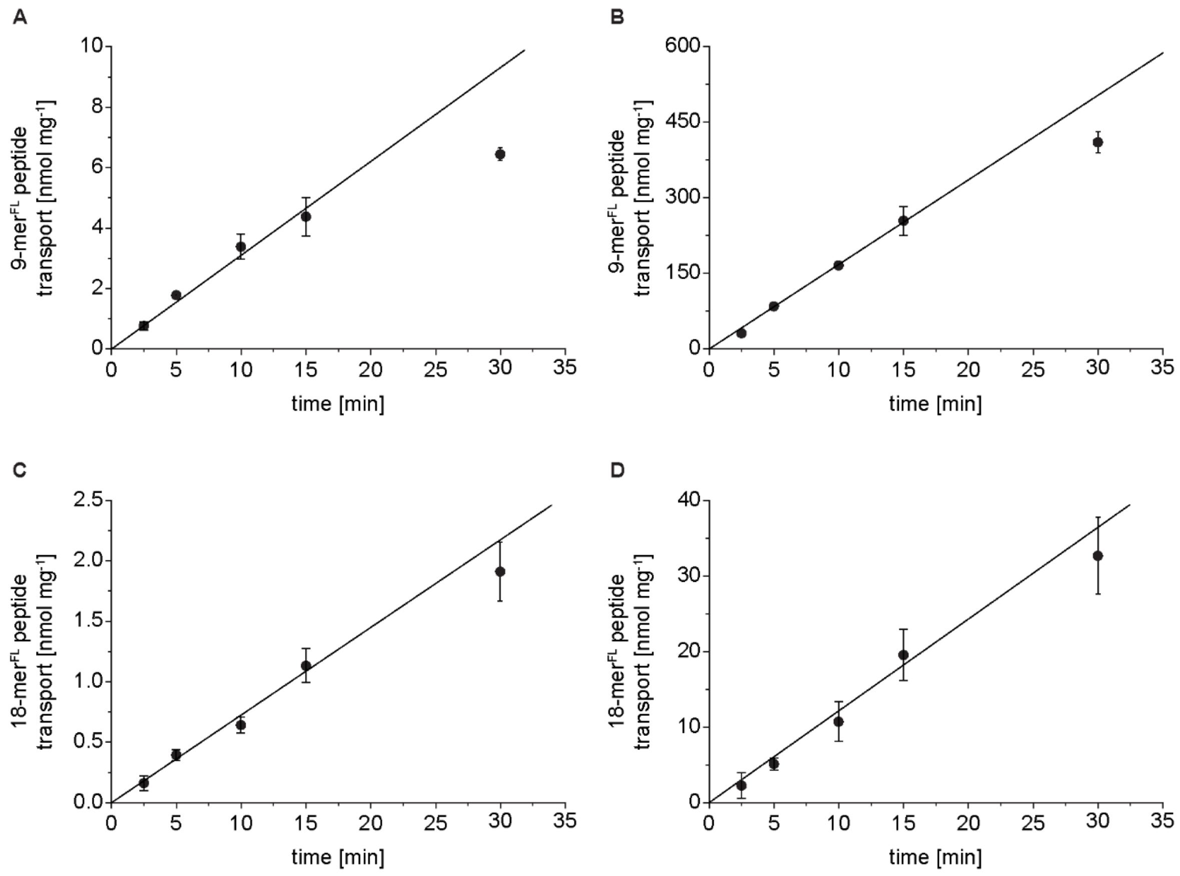

Figure S2: Time dependent peptide transport by TAPL

Transport was performed with proteoliposomes (0.5 mg/ml lipids) containing TAPL (40:1 weight ratio of lipids to TAPL) up to 30 min at 37 °C with 3 mM ATP in the presence of 0.3 (**A** and **C**) or 30 µM (**B** and **D**) peptide. Time dependent peptide transport of 9-mer^{FL} peptide (**A** and **B**) and 18-mer^{FL} peptide (**B** and **C**) was fitted with a linear regression, data points at 30 min were excluded from the fit. Transport was performed in triplicates with error bars indicating SD.

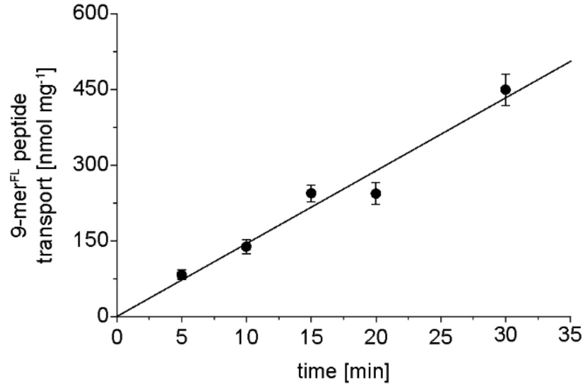

Figure S3: Time dependent 9-mer^{FL} peptide transport by TAPL at 42 °C

9-mer^{FL} peptide (100 µM) transport was performed with proteoliposomes (0.5 mg/ml lipids) containing TAPL (40:1 weight ratio of lipids to TAPL) up to 30 min at 42 °C with 3 mM ATP. Time dependent peptide transport was fitted with a linear regression, transport was performed in triplicates with error bars indicating SD.

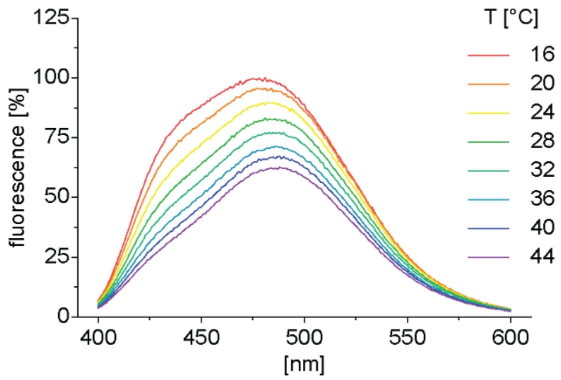

Figure S4: Emission spectra of proteoliposomes containing C-Laurdan

Proteoliposomes (33.3 µg/ml lipids) were mixed with C-Laurdan in a molar ratio of 1:500 and emission spectra were recorded between 400 and 600 nm after excitation at 375 nm for temperatures between 16 and 44 °C.

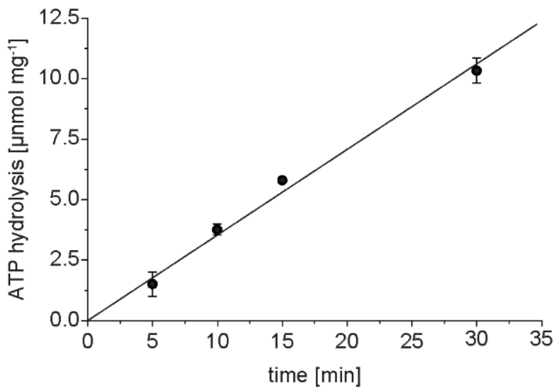

Figure S5: Time dependent ATP hydrolysis of TAPL

ATP hydrolysis was determined with proteoliposomes (0.5 mg/ml lipids) containing TAPL (40:1 weight ratio of lipids to TAPL) up to 30 min at 37 °C with 3 mM ATP by colorimetric malachite green based assay in the absence of peptide. Time dependent ATP hydrolysis was fitted with a linear regression. ATP hydrolysis was performed in triplicates with error bars indicating SD.

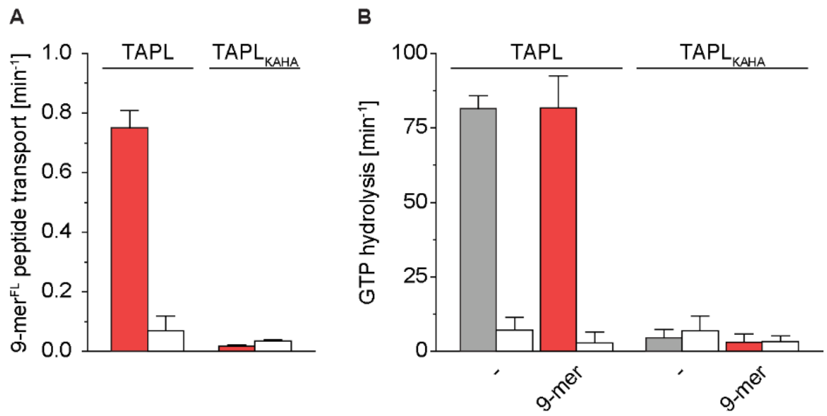

Figure S6: GTP dependent peptide transport and hydrolysis of TAPL

Proteoliposomes (0.5 mg/ml lipids) containing TAPL or $TAPL_{KAHA}$ (40:1 weight ratio of lipids to TAPL) were incubated with 3 mM GTP for 15 min at 37 °C in the absence (filled bar) or presence of ortho-vanadate (500 µM, white bar). Peptide transport (**A**) was performed in the presence of 9-mer^{FL} peptide (10 µM). GTP hydrolysis (**B**) was determined by colorimetric malachite green based assay in the absence or presence of 9-mer peptide (100 µM). All experiments were performed in triplicates, error bars indicate SD.

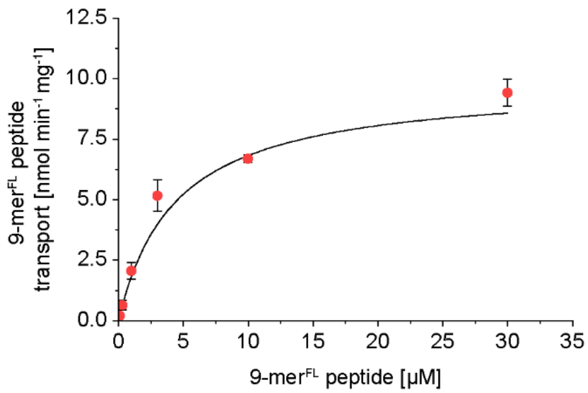

Figure S7: 9-mer^{FL} peptide transport by TAPL with GTP

Proteoliposomes (0.5 mg/ml lipids) containing TAPL (40:1 weight ratio of lipids to TAPL) were incubated for 15 min at 37 °C with rising concentrations of 9-mer^{FL} peptide in the presence of 3 mM GTP. Data were fitted by Michaelis-Menten equation (equation 4) resulting in $K_{m(Pep)}$ of 4.4 ± 0.8 µM (R^2 = 0.96). Peptide transport was performed under steady state conditions in triplicates, error bars indicate SD.

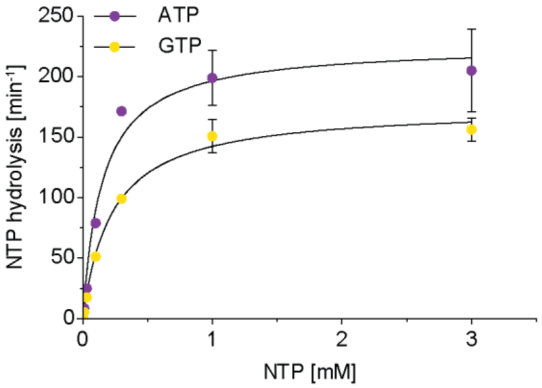

Figure S8: NTP dependent hydrolysis of TAPL

NTP hydrolysis was performed with proteoliposomes (0.5 mg/ml lipids) containing TAPL (40:1 weight ratio of lipids to TAPL) for 15 min at 37 °C in the absence of peptide. NTP hydrolysis was quantified by radioactive based assay. Data were fitted by Michaelis-Menten equation (equation 4), $K_{m(NTP)}$ and $k_{cat(NTP)}$ values are listed in Table 3. All experiments were performed in triplicates, error bars indicate SD.

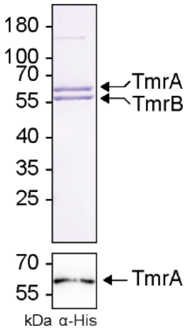

Figure S9: Purity of TmrAB

Reconstituted TmrAB was analyzed by SDS-PAGE (10%) followed by Coomassie staining (top, 2 µg protein per lane) or immunoblotting with His-tag specific antibody (bottom, 0.2 µg protein per lane). Original, uncropped Coomassie stained SDS-PAGE and immunoblots are shown in supplementary information page 13.

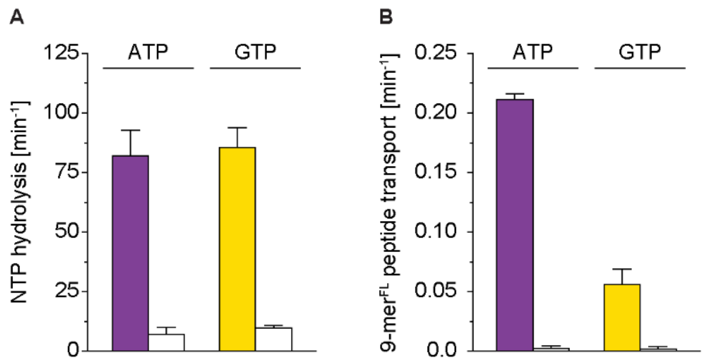

Figure S10: NTP dependent peptide transport and hydrolysis of TmrAB

Proteoliposomes (0.5 mg/ml lipids) containing TmrAB (40:1 weight ratio of lipids to TmrAB) were incubated with 3 mM ATP or GTP in the presence of 9-mer^{FL} peptide (3 µM) for 10 min at 68 °C. NTP hydrolysis determined by colorimetric malachite green based assay (**A**) and peptide transport (**B**) were performed in the absence (filled bar) or presence of ortho-vanadate (500 µM, white bar). All experiments were performed in triplicates, error bars indicate SD.

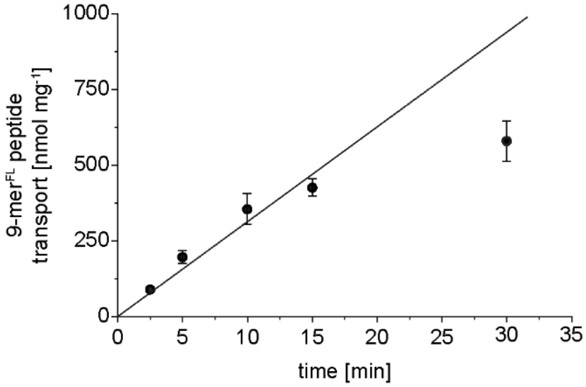

Figure S11: Time dependent peptide transport by TmrAB

Transport was performed with proteoliposomes (0.5 mg/ml lipids) containing TmrAB (40:1 weight ratio of lipids to TmrAB) up to 30 min at 68 °C with 3 mM ATP in the presence of 30 µM 9-mer^{FL} peptide. Time dependent peptide transport was fitted with a linear regression, data point at 30 min was excluded from the fit. Transport was performed in triplicates with error bars indicating SD.

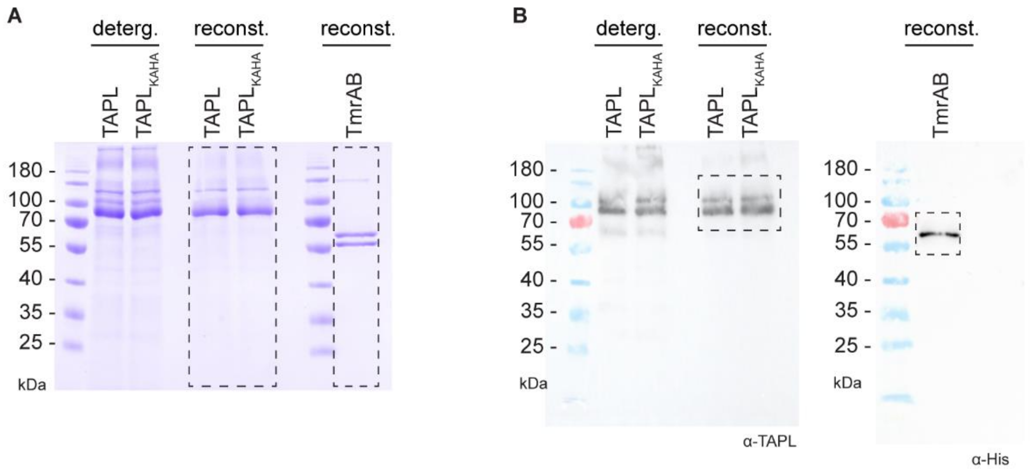

Original and uncropped Coomassie stained SDS-PAGE (**A**) and immunoblots (**B**) of Figure S1 and and S9. Dashed lines highlight areas depicted in Figure S1 and S9. Deterg. purified TAPL in detergent. Reconst. TAPL and TmrAB reconstituted in proteoliposomes.
